# National launch of human papillomavirus (HPV) immunization program in Poland, 2023

**DOI:** 10.1016/j.jvacx.2024.100436

**Published:** 2024-02-01

**Authors:** Irmina Maria Michalek, Paweł Koczkodaj, Joanna Didkowska

**Affiliations:** a Polish Onco-Hematology Registry, Polish National Cancer Registry, Maria Sklodowska-Curie National Research Institute of Oncology, Warsaw, Poland; b Department of Cancer Epidemiology and Primary Prevention, Maria Sklodowska-Curie National Research Institute of Oncology, Warsaw, Poland

**Keywords:** HPV immunization, National vaccination program, Cancer prevention, HPV-related diseases, Public health initiative, Vaccine accessibility

## Abstract

Poland launched a nationwide Human Papillomavirus (HPV) immunization program in June 2023, transitioning from a recommended to publicly funded approach. Targeting mainly 12 to 13-year-olds, the program offers universal and cost-free vaccinations with Cervarix and Gardasil 9. The initiative, aligned with the National Oncology Strategy, involves 4945 healthcare facilities, ensuring accessibility across regions. The streamlined process, empowering parents to choose, includes diverse healthcare professionals.

## Human papillomavirus (HPV) vaccination introduction in Poland

In response to the urgent need for enhanced preventive measures against HPV-related diseases, Poland initiated a pivotal step by incorporating HPV vaccination into its national immunization program starting from June 1, 2023 [1]. This marked a significant transition, as, between 2006 and this date, the HPV vaccine was authorized for distribution and recommended but not publicly funded [2]. In that period, HPV vaccines were relatively expensive, and the lack of widespread reimbursement significantly limited vaccination coverage. The average monthly salary in Poland in 2006 was 2,477 PLN gross [3], while the cost of a full vaccination was approximately 1,500 PLN [4]. Bottom-up efforts were undertaken - local government vaccination programs against HPV were implemented, but in most cases, they were exclusively intended for girls, and their implementation scale remained insufficient. For instance, in the Mazovian Voivodeship, the most populous region in Poland (population of around 5.5 million) [5], only 12 local subregions funded an HPV vaccination program, with only one of them also targeting boys (as of June 1, 2021) [6].

The universal and cost-free HPV vaccination program targets girls and boys aged 12 and 13, aligning with the objectives of the National Oncology Strategy for the years 2020–2030. Vaccination decisions are made individually after assessment by a qualified medical professional. In 2023, children born in 2010 and 2011 are eligible for vaccination, with those born in 2010 commencing the vaccination before turning 14 and completing it after reaching 15. Additionally, children previously initiated into vaccination before the program's launch can receive subsequent doses at no cost. Under the national program, two HPV vaccines, the 2-valent Cervarix (GlaxoSmithKline Biologicals s.a.) and the 9-valent Gardasil 9 (Merck Sharp & Dohme B.V.), are freely available. Both vaccines, by the Transparency Council of the Agency for Health Technology Assessment and Tariffication, effectively prevent precancerous lesions and cervical cancer [7]. Additionally, since September 2023, free HPV vaccinations have been available for children and adolescents aged 9–18, with the expanded age group applying only to the 2-valent vaccine. Furthermore, according to the Ministry of Health's information regarding the inclusion of the human papillomavirus (HPV) vaccine in the list of reimbursed drugs, as of November 1, 2023, patients above 18 years of age can receive the Cervarix vaccine at half the price (50 % reimbursement) [8]. The vaccinations consist of two doses, administered with an interval of 6 to 12 months, with the requirement to use the same vaccine for the entire regimen. The procurement of HPV vaccines is overseen by the Ministry of Health [8].

As of November 17, 2023, there are 4945 healthcare facilities across Poland offering HPV vaccinations within the program [9]. The service is available at providers with agreements related to basic health care, regardless of the declaration of primary care physician choice. Notably, the second vaccination can be administered by a different provider within the country.

The streamlined process for HPV vaccination, devoid of the need for referrals, ensures accessibility. Interested individuals can schedule appointments through various channels, including Primary Health Care clinics, the 989 hotline (operational seven days a week from 7:00 to 20:00), or the Internet Patient Account. Parents or guardians can choose between the available vaccines, Cervarix or Gardasil 9, during the appointment scheduling. It is important to note that both vaccines offer comparable benefits in preventing genital and anal precancerous lesions and cancers associated with specific oncogenic HPV types, as stated by the Ministry of Health [1], though discussions around this claim may persist.

Qualified healthcare professionals, including doctors, paramedics, nurses, midwives, and school hygienists trained in protective vaccinations, can administer the HPV vaccine. Following vaccination, an eVaccination Card is completed by the administering healthcare provider.

At the end of August 2023, within the free HPV vaccination program, 83,782 teenagers were vaccinated. The vaccination coverage stood at 9.8 %. Among all those vaccinated, 65 % were girls, and 35 % were boys (age group 12–13) [10]. The initial three-month reported coverage is notably low, indicating potential barriers related not only to individual costs but also a lack of awareness regarding the significance of HPV vaccination before the program's commencement. Strategies to enhance HPV vaccination coverage might involve targeted public awareness campaigns emphasizing the importance of HPV vaccination. Additionally, the consideration of a school-based program is prudent, although careful attention is required to address potential challenges in implementation, including logistical concerns, consent procedures, and parental apprehensions. It is noteworthy that, in contrast to the HPV vaccine, other vaccines offered to this age group, including those for flu, chickenpox, hepatitis A, and tick-borne encephalitis, are not fully covered by the country and publicly available statistics for a comparative coverage analysis are absent.

## Contextualizing HPV-related tumor incidence

In assessing the impact of HPV vaccination, it is crucial to consider the broader context of incidence rates for HPV-related tumors. According to the Polish National Cancer Registry, the 2019 age-standardized (Eurostat 2013) incidence rates, the latest available pre-COVID-19 pandemic cancer statistics, were as follows: malignant neoplasms of the cervix uteri had an incidence of 11.94 cases per 100,000, female oropharynx (defined as ICD-10: C01, C09, and C10) had an incidence of 1.32 per 100,000, and male oropharynx had an incidence of 5.27 per 100,000. While it is expected that countries with high vaccination coverage will experience a decline in incidence rates for these tumors, the current vaccination coverage in Poland suggests that without proactive measures, substantial changes in these metrics may be limited. Notably, the relatively high pap smear coverage, with 86.7 % of Polish women participating in Pap tests in 2019 [11], implies that there may be limited room for reducing cervical cancer incidence from this perspective.

## Discussion and conclusions

The implementation of a nationwide, publicly funded HPV immunization program in Poland signifies a commendable stride towards comprehensive preventive healthcare. The inclusive approach, targeting both genders and emphasizing accessibility through various healthcare providers, aims to maximize coverage. The option for parents to choose between two available vaccines, Cervarix and Gardasil 9, adds a layer of shared decision-making to increase vaccine uptake [12].

However, discussions persist around the comparable benefits of both vaccines, particularly in preventing genital and anal precancerous lesions and cancers associated with specific oncogenic HPV types [13]. As per the pronouncement by the Transparency Council of the Agency for Health Technology Assessment and Tariffication, “both available HPV vaccines in Poland exhibit effectiveness in preventing cervical cancer. In the absence of substantiated evidence showcasing clinical superiority with regard to clinically significant endpoints for either vaccine, the selection of the vaccine should be contingent upon considerations of cost and resultant economic efficiency [7].” It is noteworthy that neither the Agency nor the Ministry of Health has endorsed any study aimed at generating presently unavailable data concerning HPV types associated with cervical screening abnormalities in Poland. Particularly in the context of the aforementioned economic efficiency, it remains unclear why there has been no consideration of a single-dose HPV program. According to the World Health Organization, a one-dose HPV vaccine offers robust protection against cervical cancer [14] and has been successfully implemented by countries such as Australia [15]. Clarification and continued monitoring of these aspects are essential for maintaining public confidence in the program. Additionally, it would be advantageous for the Ministry of Health to transparently delineate the monitoring and evaluation framework subsequent to the implementation of publicly funded vaccines on a national scale. In accordance with the aforementioned, this comprehensive plan should encompass an assessment of the effects on the cervical screening program in the short to medium term, as well as an examination of the long-term impact on rates of cervical, anal, oro-pharyngeal cancers.

The involvement of a diverse range of healthcare professionals in vaccine administration, along with the simplified appointment scheduling process, underscores the program's commitment to access more remote regions and aims to maximize coverage. The completion of the eVaccination Card by administering healthcare providers ensures accurate and centralized record-keeping, contributing to the program's overall effectiveness assessment.

In conclusion, Poland's proactive approach to HPV immunization represents a noteworthy public health initiative, especially considering its position as one of the last countries in the EU to introduce cost-free HPV vaccinations [16]. This unique circumstance offers a valuable opportunity for scientific scrutiny and comparison, fostering collaboration and knowledge exchange among European nations. Continuous evaluation and communication will be crucial for addressing emerging concerns and optimizing the program's impact on reducing HPV-related diseases nationwide.

**Sources of funding:**

This research received no specific grants from any funding agency in the public, commercial, or not-for-profit sectors.

**Data sharing:**

Data analyzed in this study were a re-analysis of existing data, which are openly available at locations cited in the text.

**Author contributions:**

Drafting of the manuscript: IMM;

Critical revision of the manuscript for important intellectual content: all authors.

## CRediT authorship contribution statement

**Irmina Maria Michalek:** Writing – review & editing, Writing – original draft, Project administration, Conceptualization. **Paweł Koczkodaj:** Writing – review & editing. **Joanna Didkowska:** Writing – review & editing.

## Declaration of competing interest

The authors declare that they have no known competing financial interests or personal relationships that could have appeared to influence the work reported in this paper.

## Data Availability

All data used in this article are publicly available.
